# Cardiovascular Health in Children and Adolescents With Congenital Adrenal Hyperplasia Due to 21-Hydroxilase Deficiency

**DOI:** 10.3389/fendo.2019.00212

**Published:** 2019-04-11

**Authors:** Nicola Improda, Flavia Barbieri, Gian Paolo Ciccarelli, Donatella Capalbo, Mariacarolina Salerno

**Affiliations:** ^1^Pediatric Section, Department of Translational Medical Sciences, Federico II University of Naples, Naples, Italy; ^2^Department of Pediatrics, Federico II University of Naples, Naples, Italy

**Keywords:** Congenital Adrenal Hyperplasia, cardiovascular risk factors, 21-hydroxilase deficiency, excess androgens, obesity, cardiovascular disease

## Abstract

Increasing evidence indicates that adults with Congenital Adrenal Hyperplasia (CAH) may have a cluster of cardiovascular (CV) risk factors. In addition, ongoing research has highlighted that children and adolescents with CAH are also prone to developing unfavorable metabolic changes, such as obesity, hypertension, insulin resistance, and increased intima-media thickness, which places them at a higher risk of developing CV disease in adulthood. Moreover, CAH adolescents may exhibit subclinical left ventricular diastolic dysfunction and impaired exercise performance, with possible negative consequences on their quality of life. The therapeutic management of patients with CAH remains a challenge and current treatment regimens do not always allow optimal biochemical control. Indeed, overexposure to glucocorticoids and mineralocorticoids, as well as to androgen excess, may contribute to the development of unfavorable metabolic and CV abnormalities. Long-term prospective studies on large cohorts of patients will help to clarify the pathophysiology of metabolic alterations associated with CAH. Meanwhile, further efforts should be made to optimize treatment and identify new therapeutic approaches to prevent metabolic derangement and improve long-term health outcomes of CAH patients.

## Introduction

Deficiency of 21-hydroxylase (21-OH) is an autosomal recessive disease accounting for about 95% of the cases of Congenital Adrenal Hyperplasia (CAH). It has an incidence estimated at 1/10000-1/20000 live newborns (1). Deletions or mutations of the *CYP21A2* gene, encoding the 21-OH enzyme, impair the enzyme activity at a variable extent, resulting in glucocorticoid and/or mineralocorticoid deficiency, which lead to increased secretion of ACTH, adrenal hyperplasia, and excess production of androgens and steroid precursors before the enzymatic block (1). Based on the residual enzyme activity, CAH shows a continuum of phenotypes, ranging from a severe classic form, usually presenting in infancy, to a non-classic (NC) form, which may be diagnosed from childhood to adult life, because of signs of excess androgens production. The classic form is sub-classified as either salt-wasting (SW) or simple virilising (SV) form, depending on the degree of residual enzyme activity, which influences the amount of mineralocorticoids produced by the adrenal cortex.

Current treatment consists of glucocorticoid and, when necessary, mineralocorticoid substitution to prevent adrenal crises and to suppress the excess androgen production (2). Following the introduction of glucocorticoid treatment in the 1950s, survival of CAH patients has dramatically improved, so that CAH is now recognized as a lifelong chronic disease. However, despite recent advances in its management (i.e., prenatal diagnosis, neonatal screening, improved knowledge on adrenal medulla dysfunction) CAH still has relevant morbidity and mortality (1–4).

The therapeutic spectrum of glucocorticoids is narrow, and patients need an accurate and tailored follow-up to prevent both under- and over-treatment with glucocorticoids and/or mineralocorticoids, which may increase the cardiovascular (CV) risk of CAH patients.

Increasing evidences suggest that adult patients with CAH have a higher risk to develop long-term health problems including cardiovascular diseases, impaired fertility and bone health (3–5). However, signs and symptoms of forerunner conditions of adult disease are already detectable in childhood (6, 7).

The aim of this review is to summarize current data available on traditional and non-traditional CV risk in children and adolescents with CAH.

## Methods

We searched the PubMed database from the National Library of Medicine using the keywords “obesity,” “body composition,” “hypertension,” “lipids,” “dyslipidemia,” “glucose intolerance,” “insulin,” “insulin resistance,” “endothelial function,” “Intima-Media Thickness,” “adipokines” “inflammatory cytokines,” “blood pressure,” “cardiac morphology,” “cardiac function” associated with “Congenital Adrenal Hyperplasia,” with the limits set to only English-language articles and those involving human subjects. We included all case-control studies, case series and meta-analysis that were published in English from 1992 to date. We excluded studies involving <6 patients.

## Traditional CV Risk Factors

### Obesity and Body Composition

Several studies from large National cohorts (4, 8–10) indicate that obesity is common in adults with CAH, ranging from 22 to 52% of the subjects (9, 10). An increased prevalence of obesity has also been reported in children with CAH in most studies (8, 11–24) (Table 1), although these studies are limited by small patient number and heterogeneity of cohorts and study design. So far, only 3 relatively large cross-sectional studies reporting prevalence of obesity in CAH children are available (8, 16, 25).

**Table 1:** Summary of main studies documenting the prevalence of obesity and/or altered body composition in CAH patients.

**Study**	**Patients and CAH form**	**Age range (years)**	**Mean HC dose (mg/m^**2**^/day)**	**Mean FC dose (μg/m^**2**^/day)**	**Technique**	**Outcomes**
Finkielstain et al. (8)	-139 Classic (97 SW, 42 SV) -31 Non-classic	0.6–17	15	n.a.	n.a.	↑ Prevalence of obesity (35%) vs. normal pediatric population (17%), with no difference between Classic and NC CAH; No difference between males and females ↑ HOMA-IR ↑ BP (about 40% Classic and 20% NC CAH)
Gonçalves et al. (11)	-28 Classic females (18 SW, 10 SV) -112 Controls	4–23	15–20	n.a.	BIA	↑ BMI vs. controls, ↑ fat mass and waist/hip ratio vs. controls
Stikkelbroeck et al. (12)	-27 Classic (24 SW, 3 SV) -30 Controls	17–25	n.a.	n.a.	DXA	↑ BMI vs. controls, ↑ fat mass vs. controls
Völkl et al. (16)	-89 Classic (78 SW, 11 SV)	0.2–17.9	14.7	63.1	n.a.	↑ Prevalence of obesity (16.8%) vs. normal population (2.27%) No difference between males and females
Roche et al. (21)	-38 Classic (SW)	6.1–18.2	17.5 (prepubertal) 17.3 (pubertal) 17.8 (postpubertal)	120 (prepubertal) 113 (pubertal) 103 (postpubertal)	n.a.	↑ BMI vs. normal population ↑ SPB and DBP vs. normal population ↓ nocturnal dip in BP No difference between males and females
Subbarayan et al. (25)	-107 Classic (79 SW, 28 SV)	0.4-20.5	13.3	102	n.a.	↑ Prevalence of obesity (23.6%) vs. normal population (14.8-17.1 %) ↑ SBP vs. normal population 9.5% high plasma triglycerides No difference between males and females ↓ HOMA-IR vs. controls
Isguven et al. (26)	-16 Classic (SW) -18 Controls	1.4–10.5	15	n.a.	BIA	↑ BMI vs. controls, No difference between males and females ↑ Fat mass in female patients vs. controls
Janus et al. (27)	-61 Classic (51 SW, 10 SV) -9 Non-classic	3–17.9	17.2 (SW) 19.5 (SV) 11.9 (NC)	66.5 (SW) 28.6 (SV)	BIA	Normal BMI, no differences in body composition between classic and NC forms; No difference between males and females No hypertension
Williams et al. (28)	-25 Classic -12 Non -classic -41 Controls	0.5–15.8	13.9 8.2	82 μg/day 18 μg/day	DXA	↑ fat mass in classic CAH vs. controls, ↑ lean mass in NC CAH vs. controls Comparable HOMA-IS between CAH (classic and NC) and controls ↑ stimulated glucose and insulin in NC CAH vs. controls Comparable BP and lipids between classic CAH and controls ↑ SBP in NC CAH vs. controls
Abd El Dayem et al. (29)	-28 Classic (27 SW, 1 SV) -11 Controls	3–18	15.5	50–200 μg/day	DXA	↑ BMI, ↑ fat mass and ↓ lean mass vs. controls No difference between males and females
Marra et al. (30)	-20 Classic (15 SW, 5 SV) -20 Controls	11.1–16.1	15	54.8	DXA	↑ BMI and ↑ fat mass and waist/height ratio vs. controls; ↑ HOMA-IR vs. controls; No difference between males and females Comparable lipids and BP between CAH and controls ↓ Exercise capacity vs. controls LV diastolic dysfunction in CAH male patients
Ariyawatkul et al. (31)	-21 Classic (10 SW, 11 SV) -21 Controls	9–28	21.4	50–150 μg/day	DXA	BMI, fat and lean mass comparable between CAH and controls ↑ Waist/hip and waist/height ratio vs. controls Comparable HOMA, BP, lipids, leptin between CAH and controls
Halper et al. (32)	-32 Classic (19 SW, 13 SV) -10 Non- classic -101 controls	7.6–17.7	11.3	n.a.	DXA	↓ BMI vs. controls, VAT comparable between patients and controls
Kim et al. (33)	-28 Classic (20 SW, 8 SV) -28 Controls	12.4–18.8	19.5	210 μg/day	CT	60.7% of patients obese or overweight; ↑ VAT, SAT, and VAT/SAT vs. controls; No difference between males and females Elevated HOMA in 18% of CAH
Mooij et al. (23)	-26 Classic -(24 SW, 2 SV)	8-16	11.2	98.5	n.a.	↑ BMI vs. reference population (25.9% obese and 14.8% overweight) 44.4% of patients HOMA-IR > 75th and 29.6% HOMA-IR >90th percentile ↑ SBP in 18.5%, lower than reference population 48.1% ↓ nocturnal dip
Amr et al. (24)	-32 Classic (24 SW, 8 SV) -32 Controls	3-17	15	50-100	n.a.	↑ BMI in patients (22% obese) vs. controls ↑ HOMA-IR, CIMT, SBP vs. controls ↑ Stimulated glucose levels vs. controls Comparable lipids, stimulated insulin vs. controls

*CAH, Congenital Adrenal Hyperplasia; HC, hydrocortisone; FC, fludrocortisones; SW, salt wasting; SV, simple virilizing; NC, non-classic; HOMA, homeostatic model assessment; HOMA-IS, HOMA insulin sensitivity; BP, blood pressure; BIA, bioelectrical impedance analysis; BMI, body mass index; DXA, dual X-ray absorptiometry; SBP, systolic blood pressure; DBP, diastolic blood pressure; HOMA-IR, HOMA insulin resistance; LV, left ventricle; HDL, high density lipoproteins; T-COL, total cholesterol; VAT, visceral adipose tissue; SAT, subcutaneous adipose tissue; CIMT, carotid intima media thickness; CRP, C reactive protein. n.a., not available/not applicable. ↑ increased; ↓ decreased*.

In a study involving 170 patients, age range 0.6–17 years, about 35% of children were obese, regardless of the clinical CAH form and the glucocorticoids dose (8). Obese children presented significantly greater concentrations of insulin, leptin, and androstenedione compared to normal and overweight children. Only one patient fulfilled the diagnostic criteria for the metabolic syndrome.

Higher prevalence of obesity (16.8%) compared to the reference population (2.27%) was also reported in a cross-sectional retrospective study on 89 classic CAH patients aged 0.2–17.9 years (16). In this cohort, body mass index (BMI) positively correlated with age, hydrocortisone dose and parental BMI.

Finally, Subbarayan et al. (25) documented a prevalence of obesity of 23.6% among a cohort of 107 classic CAH patients aged 0.4 to 20.5 years. Of note, mean BMI was lower than that reported in a previous health survey from the same center almost 10 years before (21), possibly due to the use of lower glucocorticoid doses in recent years.

Whether the increased prevalence of obesity results from an increased lean body mass (as a possible consequence of excess androgens) or total/regional fat mass has been explored through bioimpedance analysis (11, 26, 27), dual x-ray absorptiometry (DEXA) (12, 15, 28–32), and computer tomography (CT) imaging (33) (Table 1). Several studies relying on DEXA documented that either males or females with classic CAH exhibit higher total fat mass (12, 15, 28–30) and reduced lean body mass (29) than controls. Moreover, some studies also documented higher indexes of visceral adiposity, such as waist/hip (11, 15) and waist-to-height ratio (WHtR) (30) with respect to controls.

Only a few data are available regarding NC CAH (27, 28, 32), documenting no significant differences between classic and NC forms. Interestingly, one study reported a higher lean body mass in NC CAH in comparison to matched controls, suggesting an effect of longer exposure to excess androgens (28).

A weak correlation between 6-months cumulative glucocorticoid dose and total fat mass was found only in females (12), whereas duration of treatment was positively correlated to the percentage of total fat mass and to fat/lean ratio (29).

In contrast to these results, two recent DEXA studies failed to identify significant differences in body composition between CAH patients and controls (31, 32). One of these studies (32) used DEXA to estimate visceral adipose tissue (VAT), which was also comparable between CAH patients and controls.

To date, only one study performing CT imaging is available, revealing greater amount of both VAT and subcutaneous adipose tissue (SAT) and VAT/SAT ratio in 28 adolescents and young adults with classic CAH, compared with age-, gender-, and BMI-matched controls (33). SAT and VAT were positively correlated to adiposity indexes (BMI, WHtR, trunk, and total body fat mass), homeostasis model assessment (HOMA), and lipid profile (total cholesterol, low density lipoproteins (LDL), very low density lipoproteins (VLDL) and triglycerides), but did not correlate with hydrocortisone dose or markers of hormonal control.

Interestingly, adiposity rebound, consisting in the re-increase of BMI after its nadir, occurs significantly earlier in CAH patients than non-affected children, possibly predicting development of obesity (15, 34). In a recent study (35) on 29 classic CAH patients identified by neonatal screening and followed-up for at least 10 years, multivariate regression analysis identified lower BMI at birth as an independent predictor of early adiposity rebound, thus suggesting that early alterations in fetal life may predispose to the development of metabolic problems.

In conclusion, current data point toward a tendency to develop increased adiposity in children and adolescents with CAH. Supra-physiological dosages of glucocorticoids have been advocated as a key causative factor, however, data regarding this association are not univocal, and thus other predisposing factors may contribute to an increased risk of obesity.

Even though obesity has potential negative effects in terms of cardiac and metabolic consequences, recent Endocrine Society Guidelines on CAH (2) recommend against routine evaluation for cardiac and metabolic disease in patients with CAH beyond that advised for the general population, and suggest early lifestyle counseling in order to prevent such consequences.

### Insulin Resistance

Several studies have demonstrated a tendency toward insulin resistance (IR) in children and adolescents with CAH, as assessed by increased plasma insulin concentrations (30) and HOMA-IR (8, 23, 24, 30, 33, 36–38) (Table 2).

**Table 2:** Summary of main studies addressing insulin-sensitivity in CAH patients.

**Study**	**Patients and CAH form**	**Age range (years)**	**Mean HC dose (mg/m^**2**^/day)**	**Mean FC dose(μg/m^**2**^/day)**	**Methods**	**Outcomes**
Finkielstain et al. (8)	-139 Classic (97 SW, 42 SV) -31 Non-classic	0.6–17	15	n.a.	n.a.	↑ Prevalence of obesity (35%) vs. normal pediatric population (17%), with no difference between Classic and NC CAH and between males and females↑ HOMA-IR↑ BP (about 40% Classic and 20% NC CAH)
Marra et al. (30)	-20 Classic (15 SW, 5 SV) -20 Controls	11.1–16.1	15	54.8	HOMA	↑ BMI and ↑ fat mass and waist/height ratio vs. controls; ↑ HOMA-IR vs. controls; No difference between males and females Comparable lipids and BP between CAH and controls ↓ exercise capacity vs. controls LV diastolic dysfunction in CAH male patients
Kim et al. (33)	-28 Classic (20 SW, 8 SV) -28 Controls	12.4–18.8	19.5	210 μg/day	HOMA	60.7% of patients obese or overweight; ↑ VAT, SAT and VAT/SAT vs. controls; No difference between males and females Elevated HOMA in 18% of CAH
Charmandari et al. (36)	-16 Classic (12 SW, 4 SV) -28 Controls	2–12	14.8	114 μg/day	HOMA	BMI comparable to controls ↑ HOMA-IR vs. controls ↑ Leptin in patients vs. controls; No difference between males and females
Harrington et al. (37)	-14 Classic (11 SW, 3 SV) 53 Controls	11.6–18	13.3	108.3 μg/day	HOMA	BMI comparable to controls, ↑ WHtR vs. controls ↑ HOMA-IR vs. controls ↑ SBP vs. controls ↓ FMD and smooth muscle function vs. controls IMT, CRP, lipids comparable between CAH and controls
Mooij et al. (23)	-26 Classic (24 SW, 2 SV)	8–16	11.2	98.5	HOMA	↑ BMI vs. reference population (25.9% obese and 14.8% overweight) 44.4% of patients HOMA-IR > 75th and 29.6% HOMA-IR >90th percentile ↑ SBP in 18.5%, lower than reference population 48.1% ↓ nocturnal dip
Amr et al. (24)	-32 Classic (24 SW, 8 SV) -32 Controls	3–17	15	50–100	HOMA OGTT	↑ BMI in patients (22% obese) vs. controls ↑ HOMA-IR, CIMT, SBP vs. controls ↑ Stimulated glucose levels vs. controls Comparable lipids, stimulated insulin vs. controls
Zimmermann et al. (38)	-27 Classic (12 SW, 15 SV) -27 Controls	4–31	21.5 (SW) 16.2 (SV)	50–100 μg/day	HOMA IRI ISI OGTT	BMI comparable to controls ↓ HOMA-IS and ↑ HOMA-IR, HOMA-B, IRI, stimulated glucose and insulin levels vs. controls ↑ Small dense LDL subfractions and HDL vs. controls Comparable LDL, T-COL, triglycerides between CAH and controls
Ariyawatkul et al. (31)	-21 Classic (10 SW, 11 SV) -21 Controls	9–28	21.4	50–150 μg/day	HOMA	BMI, fat and lean mass comparable between CAH and controls ↑ Waist/hip and waist/height ratio vs. controls Comparable HOMA, BP, lipids, leptin between CAH and controls
Subbarayan et al. (25)	-107 Classic (79 SW, 28 SV)	0.4–20.5	13.3	102	HOMA	↑ Prevalence of obesity (23.6%) vs. normal population (14.8–17.1%) ↑ SBP vs. normal population 9.5% high plasma triglycerides No difference between males and females ↓ HOMA-IR vs. controls
Williams et al. (28)	-25 Classic -12 Non-classic -41 Controls	0.5–15.8	13.9 8.2	82 μg/day 18 μg/day	HOMA OGTT	↑ Fat mass in classic CAH vs. controls ↑ Lean mass in NC CAH vs. controls Comparable HOMA-IS between CAH (classic and NC) and controls ↑ Stimulated glucose and insulin in NC CAH vs. controls Comparable BP and lipids between classic CAH and controls ↑ SBP in NC CAH vs. controls
Saygili et al. (39)	-18 Non-classic -26 Controls	25.7 ± 8.9 (mean ± SD)	Untreated	Untreated	HOMA	BMI comparable to controls ↑ HOMA-IR vs. controls
Bayraktar et al. (40)	-50 Non-classic -25 Controls	22.1 ± 2.91 (mean ± SD)	Untreated	Untreated	HOMA	BMI comparable to controls Comparable HOMA-IR and lipids between CAH and controls
Speiser et al. (41)	-6 Non-classic -12 Controls	16–45	Untreated	Untreated	i.v. GTT	BMI comparable to controls ↓ Insulin sensitivity vs. controls
Wasniewska et al. (42)	-9 Classic SW -9 Non-classic -16 Controls	13.3–20.4	17.1	100 μg/day	HOMA	BMI comparable to controls ↑ HOMA-IR in Classic CAH vs. controls Comparable HOMA-IR between NC CAH and controls ↑ SBP in classic CAH vs. controls ↑ DBP in classic CAH and NC CAH vs. controls Comparable lipids between CAH and controls ↑ IMT in classic CAH and NC CAH vs. controls

*CAH, Congenital Adrenal Hyperplasia; HC, hydrocortisone; FC, fludrocortisones; SW, salt wasting; SV, simple virilizing; HOMA, homeostatic model assessment; BMI, body mass index; HOMA-IR, HOMA insulin resistance; BP, blood pressure; LV, left ventricle; VAT, visceral adipose tissue; SAT, subcutaneous adipose tissue; WHtR, waist-to-height ratio; SBP, systolic blood pressure; FMD, flow-mediated dilatation; IMT, intima media thickness; CRP, C reactive protein; OGTT, oral glucose tolerance test; IRI, insulin resistance index; ISI, insulin sensitivity index; LDL, low density lipoproteins; HDL, high density lipoproteins; NC, non-classic; T-COL, total cholesterol; i.v. GTT, intravenous glucose tolerance test; DBP, diastolic blood pressure. ↑ increased; ↓ decreased*.

In a recent study, 8 out of 27 (29.6%) classic CAH patients presented a HOMA-IR above the 90th percentile, which was significantly correlated to hydrocortisone dose and BMI (23).

On the other hand, other studies have reported normal (31) or even better (25) insulin sensitivity in CAH patients compared to controls. In particular in a large cohort of 107 children and adolescents with classic CAH (25), despite increased BMI, CAH children had lower levels of fasting blood glucose, insulin and HOMA-IR compared to controls.

Glucose tolerance and insulin sensitivity status have also been assessed through oral glucose tolerance test (OGTT) (Table 2). In a recent study (24) on 32 patients with classic CAH aged 3–17 years, impaired fasting glucose was found in 34% of the patients, while 19% had impaired glucose tolerance and 2.7% a slight increase in HOMA-IR. Noteworthy, in this study BMI was significantly higher than controls, with 22% of the patients labeled as obese. However, other studies suggest a tendency toward IR even in the absence of obesity (28, 38). Zimmerman et al. (38) performed OGTT in 27 normal weight children and young adults with classic CAH showing higher post-load glucose and insulin values than matched controls. Moreover, insulin resistance index (IRI) and HOMA-insulin secretion B cell (HOMA-B) were found to be higher and insulin sensitivity index (ISI) to be lower in patients compared with controls. IRI was significantly correlated with hydrocortisone dose and duration of treatment (38).

With regard to NC CAH, only a few data on small cohorts are available (28, 39–42). Most of the studies documented that both treated (28, 42) and untreated (39, 41) NC CAH patients have reduced insulin sensitivity, thus suggesting that prolonged exposure to androgen excess before diagnosis may contribute to IR. Williams et al. (28) documented that, despite more favorable body composition, NC CAH had more pronounced alterations in glucose metabolism compared to classic forms.

Finally, the results of a recent meta-analysis of 14 studies, reporting data for 437 children and adolescents with CAH, confirm that CAH patients display reduced insulin sensitivity compared to controls (6).

In summary, several data suggest that children and adolescents with CAH have impaired insulin sensitivity, with preserved glucose tolerance. Obesity and altered body composition are risk factors for IR; however, unfavorable changes in glucose metabolism may also be detected in non-obese CAH subjects. Indeed, glucocorticoid excess and hyperandrogenism may both contribute to the development of IR.

### Dyslipidemia

Dyslipidemia may be a possible consequence of increased body fat. However, in the majority of CAH children the lipid profile is comparable to controls (24, 28, 30, 42, 43). Subtle alterations in lipid metabolism have been detected only in a few studies on small cohorts of children with several methodological limitations (38, 44). Indeed, higher triglycerides (44), lower HDL cholesterol and higher concentrations of small dense LDL subfractions (sd-LDL) (38) have been reported.

A recent study on a large cohort of 107 children with CAH aged 9.2 years (range 0.4–20.5 years) documented a slight increase in the prevalence of hyperlipidemia (9.5%), even though a proper control group to assess the significance of these findings was lacking (25).

At present, there are not sufficient data documenting dyslipidemia in CAH children, therefore, regular assessment of lipid profile seems to be unnecessary and further studies are needed to better address this topic.

### Hypertension

The vast majority of studies investigating blood pressure (BP) in CAH patients, either by ambulatory or 24 h BP measurement, documented increased systolic and/or diastolic BP values (6, 8, 21–23, 25, 43, 45–47), as summarized in Table 3. Moreover, even in the absence of overt hypertension (27), CAH children may display a reduction of the physiological nocturnal dipping in BP (21, 23, 46).

**Table 3:** Summary of main studies investigating hypertension in CAH patients.

**Study**	**Patients and CAH form**	**Age range (years)**	**Mean HC dose (mg/m^**2**^/day)**	**Mean FC dose (μg/m^**2**^/day)**	**Methods**	**Outcomes**
Roche et al. (21)	-38 Classic SW	6.1–18.2	17.5 (prepubertal) 17.3 (pubertal) 17.8 (postpubertal)	120 (prepubertal) 113 (pubertal) 103 (postpubertal)	24-h BP	↑ BMI vs. normal population ↑ SPB and DBP vs. normal population ↓ Nocturnal dip in BP No difference between males and females
Bonfig et al. (22)	-716 Classic (571 SW, 145 SV)	3–18	14.4	72.7	Morning BP	↑ BMI in SW vs. SV ↑ prevalence of hypertension in 12.5% of children ↑ BP in SW vs. SV More elevated SBP than DBP At the pubertal age↑prevalence of hypertension in females than in males (12 vs. 5.3%)
Subbarayan et al. (25)	-107 Classic (79 SW, 28 SV)	0.4–20.5	13.3	102	Mean of four measurements (every 6 h)	↑ prevalence of obesity (23.6%) vs. normal population (14.8–17.1%) ↑ SBP vs. normal population 9.5% high plasma triglycerides No difference between males and females ↓ HOMA-IR vs. controls
Mooij et al. (23)	-26 Classic (24 SW, 2 SV)	8–16	11.2	98.5	HOMA	↑ BMI vs. reference population (25.9% obese and 14.8% overweight) 44.4% of patients HOMA-IR > 75th and 29.6% HOMA-IR >90th percentile ↑ SBP in 18.5%, lower than reference population 48.1% ↓ nocturnal dip
Akyürek et al. (43)	-25 Classic SW -25 Controls	5–15	17.03	120	Morning BP 24-h BP	↑ BMI vs. control Comparable HOMA-IR between CAH and controls ↑ DBP vs. controls ↓ Nocturnal dip of SBP and DBP vs. controls Comparable lipids between CAH and controls ↑ IMT vs. controls
Nebesio and Eugster (45)	-91 Classic	n.a.	16.4	90 μg/day	n.a.	↑ prevalence of hypertension vs. normal population
Völkl et al. (46)	55 Classic (45 SW, 10 SV)	5.3–19	14.6	47	24-h BP	↑ BMI ↑ daytime and nighttime SBP ↓ nocturnal dip of DBP No difference between males and females in ambulatory 24-h BP
Hoepffner et al. (47)	-23 Classic	6–17	18.7	70.8	Morning BP 24-h BP	↑ SPB and DBP in hospitalized vs. outpatients No difference between males and females
Janus et al. (27)	-61 Classic (51 SW, 10SV) 9 Non-classic	3–17.9	17.2 (SW) 19.5 (SV) 11.9 (NC)	66.5 (SW) 28.6 (SV)	24-h BP	Normal BMI, no differences in body composition between classic and NC forms No overt hypertension but ambulatory 24-h SBP and Night SBP were higher in females than males
Finkielstain et al. (8)	-139 Classic (97 SW, 42 SV) -31 Non-classic	0.6–17	15	n.a.	n.a.	↑ Prevalence of obesity (35%) vs. normal pediatric population (17%), with no difference between Classic and NC CAH and between males and females ↑ HOMA-IR ↑ BP (about 40% Classic and 20% NC CAH)

*CAH, Congenital Adrenal Hyperplasia; HC, hydrocortisone; FC, fludrocortisones; SW, salt wasting; BP, blood pressure; BMI, body mass index; SBP, systolic blood pressure; DBP, diastolic blood pressure; SV, simple virilizing; HOMA, homeostatic model assessment; HOMA-IR, HOMA insulin resistance; IMT, intima media thickness; NC, non-classic; HOMA-IS, HOMA insulin sensitivity; n.a., not available/non-applicable. ↑ increased; ↓ decreased*.

Subbarayan et al. (25) reported systolic hypertension in 20.9%, diastolic hypertension in 8.8%, and both systolic and diastolic hypertension in 3% of a large cohort of CAH children, regardless of gender. However, only systolic BP (SBP) was significantly higher compared to the reference population. Interestingly, the prevalence of hypertension was lower compared to previous studies (21, 46), possibly due to the use of lower glucocorticoid and mineralocorticoid doses in recent years. Both SBP and diastolic BP (DBP) were negatively related to age, possibly due to a reduction in the dose of fludrocortisone.

A recent longitudinal multicenter study (22) on a large cohort of classic CAH patients reported a prevalence of hypertension of 12.5%. The increase in SBP was more marked than what was observed for DBP. SW patients exhibited higher BP values than SV patients. BP values were significantly correlated to BMI, age and fludrocortisone dose. Of note, the prevalence of hypertension in CAH patients decreases with age, becoming comparable to the general population (below 5%) at the age of 18 years. In keeping with this, BP values were significantly lower in a French National survey on men with classic 21-OH deficiency compared to healthy population (10), even though other studies reported elevated BP in men (8) or women (4, 9) with CAH, possibly due to differences in treatment regimens among different studies. Finally, significant alterations in BP in CAH patients have been confirmed in the recent meta-analysis by Tamhane et al. (6).

Few studies investigated gender differences, most reporting a similar prevalence of hypertension between males and females with CAH (8, 21, 25, 46, 47). Only in two studies, evaluating morning blood pressure (22) or 24-h BP monitoring (27), CAH females seemed more affected then males, likely due to excess androgen exposure.

Taken together, current findings suggest a higher prevalence of hypertension in CAH children and adolescents, with a selective increase in systolic rather than diastolic BP values. In addition to obesity, the excess of glucocorticoid and mineralocorticoid doses also play a key pathogenic role, especially in the first years of life, when higher doses and salt supplementation are often needed, in order to counteract the physiologic state of relatively higher resistance to mineralocorticoids and the higher renal salt wasting.

## Non-Traditional CV Risk Factors

### Circulating Cytokines

Adipose tissue represents an important source of inflammatory cytokines and thus, a few studies have evaluated inflammatory markers in CAH patients. A study by Charmandari et al. (36) including 18 classic CAH patients (age range 2–12 years) showed significantly higher leptin concentrations than healthy controls, regardless of BMI and sex. Leptin concentrations were negatively correlated with epinephrine and free metanephrine concentrations, and are likely related to reduced inhibitory effect of catecholamines on leptin secretion. Loss of gender dimorphism in leptin concentrations was also observed in the CAH group, possibly due to exposure to androgens excess in females.

Increased leptin concentrations and a strong correlation between leptin, obesity (8, 23, 33) and HOMA-IR (23) have also been documented in studies, enrolling both classic and NC CAH patients.

Conversely, other studies (19, 26, 31) found leptin concentrations in CAH children and adolescents comparable to controls, even in the face of higher BMI and body fat ratio (26). However, significantly lower concentrations of soluble leptin receptor (sOB-R) (which binds circulating leptin, regulating its half-life) were found in CAH patients compared to matched controls, predicting a higher amount of free (unbound) serum leptin (19).

The role of adiponectin on CV health of CAH children is still unclear. Higher adiponectin concentrations than matched controls, with no alteration in serum leptin and adiponectin/leptin ratio, were found in a study on 51 CAH patients, aged 5.6 to 19.6 years, regardless of sex (48). Adiponectin was negatively correlated with BMI, serum DHEA-S and testosterone, but no clear relationship with hydrocortisone and fludrocortisone dosage (48).

A few studies have also investigated the role of other inflammatory markers in CAH patients. In particular, high sensitivity C reactive protein (hs-CRP), interleukin-6 (IL-6), and plasminogen activator inhibitor-1 (PAI-1) have been found to be normal in two studies (31, 33), while tissue plasminogen activator (tPA) concentrations were higher than controls (23). In addition, a study by Metwalley et al. (49) demonstrated that, compared to controls, classic CAH patients (*n* = 32, mean age 13.6 ± 2.5 years) have higher concentrations of hs-CRP as well as of circulating endothelial cells (CECs), which represent an indicator of vascular endothelial injury. Both hs-CRP and CECs were correlated to poor disease control, IMT and indexes of diastolic dysfunction.

Finally, homocysteine concentrations were also higher than controls (despite comparable folate and vitamin B12 levels), especially in poorly controlled CAH patients aged 5–12 years (50).

In conclusion, a few data suggest that CAH may be associated with an unbalanced profile of adipokines, inflammatory markers and homocysteine, which are all considered early markers of CV morbidity. Further research is needed to unravel whether these alterations represent the consequence of poor disease control, unfavorable metabolic profile or are intrinsic to CAH.

### Vascular Function and Intima-Media Thickness

Intima-media thickness (IMT), a surrogate marker of atherosclerosis, has been evaluated in several studies (23, 29, 33, 42, 51), yielding contrasting results. A few studies found normal values of carotid-IMT (C-IMT), comparable to controls (23, 29, 33). However, a loss of sexual dimorphism in females exposed to excess androgens and a correlation between C-IMT, 17-hydroxyprogesterone (17-OHP), and androstenedione concentration were observed (29).

In contrast, significant differences in IMT values between CAH patients and controls have been reported in other studies (6, 24, 42, 43, 49, 51, 52) with no differences between SV and SW forms (24), or normal weight and overweight subjects (52). In the study by Akyürek et al. (43), C-IMT values were greater in hypertensive compared to normotensive CAH patients, and were negatively correlated to nocturnal dipping of DBP. A significant correlation was also found between C-IMT and indexes of disease control, such as duration of treatment, and concentrations of 17-OHP and testosterone (49), suggesting that increased androgen levels may contribute to an increased risk of vascular dysfunction. Janus and coworkers (27) evaluated arterial ambulatory stiffness index (AASI), derived by 24-h blood pressure measurement, reporting higher values in SW forms with genotypes Del/Del and Del/I2G compared to other forms, especially in females. This further suggests a detrimental role of excess androgens or treatment on arterial wall function. Indeed, AASI was related to urinary cortisol, free androgen index and bone age advancement. Positive correlations between carotid and femoral IMT with BMI (51), aortic and carotid IMT with serum triglycerides, and aortic IMT with cumulative glucocorticoid doses (42), suggest how subclinical atherogenesis in CAH children may arise from a complex interplay between overweight, dyslipidemia and hypercortisolism.

Furthermore, Özdemir et al. (53) has recently reported increased stiffness index and elastic modulus of the aorta and the carotid artery, with lower aortic and carotid distensibility in CAH children and adolescents. Multivariate regression analysis identified BMI as the only independent variable for C-IMT and aortic stiffness index.

Finally, classic CAH patients may also exhibit impaired endothelial function, evaluated by flow-mediated dilatation (FMD) and glyceryl trinitrate-mediated dilatation (GTN), in comparison to healthy controls (37). In this study, BMI was comparable between CAH children and controls, thus suggesting that other factors, beyond increased adiposity, are likely to contribute to the development of endothelial dysfunction.

Although current studies are often limited by small sample size, heterogeneous population and study designs, preliminary evidences suggest that CAH may be associated with vascular dysfunction and increased IMT already in childhood. Obesity, hypertension, glucocorticoid overtreatment, as well as prolonged exposure to androgen excess, may all contribute to an increased risk of early atherosclerosis, even though the role of each contributing factors is far from being clear.

## Cardiac Morphology and Function

Adults affected by CAH may have increased CV morbidity (4) even though the pathogenic mechanism is still unclear. Recent studies showed that alterations in cardiac morphology and function in CAH may be detected already in childhood (30, 49, 53, 54).

We recently demonstrated that classic CAH adolescent males may have signs of mild subclinical diastolic dysfunction, consisting in a significant prolongation of isovolumetric relaxation time (IVRT) and mitral deceleration time (MDT), compared to matched controls (30). These indexes of diastolic dysfunction correlated negatively with testosterone concentrations, which were, in turn, negatively related to cumulative hydrocortisone dose in the 3 years before the study. This led to the hypothesis that excess of glucocorticoid treatment during adolescence may affect cardiac function through either direct detrimental effect on muscle performance (55) or through induction of mild hypogonadism.

Our results have been confirmed by other studies (49, 53, 54). Left ventricular diastolic dysfunction, characterized by higher late diastolic myocardial velocity (Am) and significantly lower early diastolic myocardial velocity (Em)/Am ratio in comparison to controls, was documented by Özdemir et al. (53). In addition to mild diastolic dysfunction, Metwalley et al. (49) also reported higher LV mass (LVM) in classic CAH adolescents compared to matched controls, which was more pronounced in patients with clinical signs of androgen excess and high serum 17-OHP.

Conversely, normal LV mass was reported in a cohort of 20 classic CAH patients; however, no data on LV cavity size or functional data were provided and no comparison with a control group was made (56). Mooij et al. (57) recently documented, in a cohort of 27 CAH patients (mean age 11.7 years), lower IVR and LV posterior wall thickness, and higher prevalence of incomplete right bundle branch block, compared to matched controls. Shorter exposure to excess androgens (due to neonatal screening allowing early diagnosis) and/or better control of the disease in this cohort have been proposed to explain differences from previous studies (57).

Exercise capacity in CAH adolescents has been investigated in very small studies performing both short-term high-intensity (58) and long-term moderate-intensity exercise (59). In these two studies, exercise capacity and systolic BP at peak exercise were similar to controls; however, epinephrine and metanephrine concentrations at baseline and peak exercise, as well as glucose values throughout exercise and recovery, were lower in CAH patients than in healthy controls (58, 59). Although lower concentrations of epinephrine and metanephrine have been detected in CAH patients compared with healthy controls (60), quality of current evidences does not allow for identification of a clear role of adrenomedullary dysfunction in exercise performance.

Recently, we reported that adolescents affected with classic CAH (age range 13.6 ± 2.5 years) have impaired exercise capacity, with reduced peak workload and exaggerated systolic BP at peak exertion during an incremental exercise test on a bicycle ergometer (30). Unfortunately, we did not measure epinephrine and metanephrine levels, and thus, we could not evaluate the role of adrenomedullary dysfunction in impaired exercise performance in our patients. However, we documented cardiac alterations and impaired exercise capacity, similar to CAH, in a group of adolescents with juvenile idiopathic arthritis who were treated with comparable doses of glucocorticoids, thus suggesting a pathophysiological link between glucocorticoid replacement and CV abnormalities.

In summary, current evidences suggest that CAH adolescents display subclinical diastolic dysfunction, LV hypertrophy and impaired exercise performance, with possible negative consequences on their quality of life. Further studies on larger cohorts are necessary to define the mechanisms underlying these abnormalities and the role of over- and under-treatment.

## Conclusions

Increasing evidence indicates that CAH individuals are prone to develop a number of early CV risk factors, such as obesity, hypertension, insulin resistance, low-grade inflammation, increased IMT and subclinical cardiac dysfunction, already in childhood.

The therapeutic management of patients with CAH remains a challenge and current treatment regimens do not always allow optimal biochemical control. Overexposure to glucocorticoids and mineralocorticoids as well as to androgens may contribute to the development of unfavorable metabolic and CV changes, even though metabolic derangement in CAH patients may also result from other still unraveled risk factors.

At present, there is insufficient evidence to recommend regular monitoring of early metabolic markers of CV disease in all CAH children. However, lifestyle counseling to avoid overweight and other related metabolic consequences, and regular assessment of blood pressure at all ages should be recommended in the management of CAH children. Monitoring for other metabolic and CV abnormalities should be tailored to individual patient's needs.

Long-term prospective studies on large cohorts of patients are necessary to better clarify the pathophysiology of metabolic alterations associated with CAH. In the meantime, further efforts should be made in order to optimize treatment, and identify new therapeutic approaches to prevent metabolic derangement and improve long-term health outcomes of CAH patients.

## Author Contributions

NI, MS, and DC ideated the manuscript. NI, FB, GC, and DC drafted the manuscript. DC and MS revised the manuscript for intellectual contents. All authors listed have made a substantial, direct and intellectual contribution to the work, and approved it for publication.

### Conflict of Interest Statement

The authors declare that the research was conducted in the absence of any commercial or financial relationships that could be construed as a potential conflict of interest. The reviewer AC declared a shared affiliation, though no other collaboration, with the authors to the handling Editor.

## References

[1] El-Maouche D Arlt W Merke DP. Congenital adrenal hyperplasia. Lancet. (2017) 390:2194–210. 10.1016/S0140-6736(17)31431-9 28576284

[2] Speiser PW Arlt W Auchus RJ Baskin LS Conway GS Merke DP. Congenital adrenal hyperplasia due to steroid 21-hydroxylase deficiency: an endocrine society clinical practice guideline. J Clin Endocrinol Metab. (2018) 103:4043–88. 10.1210/jc.2018-01865 30272171 PMC6456929

[3] Reisch N Arlt W Krone N. Health problems in congenital adrenal hyperplasia due to 21-hydroxylase deficiency. Horm Res Paediatr. (2011) 76:73–85. 10.1159/000327794 21597280

[4] Falhammar H Frisén L Hirschberg AL Norrby C Almqvist C Nordenskjöld A. Increased cardiovascular and metabolic morbidity in patients with 21-hydroxylase deficiency: a swedish population-based national cohort study. J Clin Endocrinol Metab. (2015) 100:3520–8. 10.1210/JC.2015-2093 26126207

[5] Bachelot A Grouthier V Courtillot C Dulon J Touraine P. Management of endocrine disease: congenital adrenal hyperplasia due to 21-hydroxylase deficiency: update on the management of adult patients and prenatal treatment. Eur J Endocrinol. (2017) 176:R167–81. 10.1530/EJE-16-0888 28115464

[6] Tamhane S Rodriguez-Gutierrez R Iqbal AM Prokop LJ Bancos I Speiser PW. Cardiovascular and metabolic outcomes in congenital adrenal hyperplasia: a systematic review and meta-analysis. J Clin Endocrinol Metab. (2018) 103:4097–103. 10.1210/jc.2018-01862 30272185

[7] Mooij CF Webb EA Claahsen van der Grinten HL Krone N. Cardiovascular health, growth and gonadal function in children and adolescents with congenital adrenal hyperplasia. Arch Dis Child. (2017) 102:578–84. 10.1136/archdischild-2016-311910 27974295

[8] Finkielstain GP Kim MS Sinaii N Nishitani M Van Ryzin C Hill SC. Clinical characteristics of a cohort of 244 patients with congenital adrenal hyperplasia. J Clin Endocrinol Metab. (2012) 97:4429–38. 10.1210/jc.2012-2102 22990093 PMC3513542

[9] Arlt W Willis DS Wild SH Krone N Doherty EJ Hahner S. Health status of adults with congenital adrenal hyperplasia: a cohort study of 203 patients. J Clin Endocrinol Metab. (2010) 95:5110–21. 10.1210/jc.2010-0917 20719839 PMC3066446

[10] Bouvattier C Esterle L Renoult-Pierre P de la Perrière AB Illouz F Kerlan V. Clinical outcome, hormonal status, gonadotrope axis, and testicular function in 219 adult men born with classic 21-hydroxylase deficiency. A French National Survey. J Clin Endocrinol Metab. (2015) 100:2303–13. 10.1210/jc.2014-4124 25822101

[11] Gonçalves EM de Lemos-Marini SH de Mello MP Baptista MT D'Souza-Li LF Baldin AD. Impairment in anthropometric parameters and body composition in females with classical 21-hydroxylase deficiency. J Pediatr Endocrinol Metab. (2009) 22:519–29. 10.1515/JPEM.2009.22.6.519 19694199

[12] Stikkelbroeck NM Oyen WJ van der Wilt GJ Hermus AR Otten BJ. Normal bone mineral density and lean body mass, but increased fat mass, in young adult patients with congenital adrenal hyperplasia. J Clin Endocrinol Metab. (2003) 88:1036–42. 10.1210/jc.2002-021074 12629082

[13] Bachelot A Plu-Bureau G Thibaud E Laborde K Pinto G Samara D. Long-term outcome of patients with congenital adrenal hyperplasia due to 21-hydroxylase deficiency. Horm Res. (2007) 67:268–76. 10.1097/MAJ.0b013e31824369e4 17170529

[14] Hagenfeldt K Martin RE Ringertz H Helleday J Carlstrom K. Bone mass and body composition of adult women with congenital virilizing 21-hydroxylase deficiency after glucocorticoid treatment since infancy. Eur J Endocrinol. (2000) 143:667–71. 10.1530/eje.0.1430667 11078991

[15] Cornean RE Hindmarsh PC Brook CG. Obesity in 21-hydroxylase deficient patients. Arch Dis Child. (1998) 78:261–63. 10.1136/adc.78.3.261 9613359 PMC1717507

[16] Völkl TM Simm D Beier C Dorr HG. Obesity among children and adolescents with classic congenital adrenal hyperplasia due to 21-hydroxylase deficiency. Pediatrics. (2006) 117:e98–105. 10.1542/peds.2005-1005 16396852

[17] Helleday J Siwers B Ritzén EM Carlström K. Subnormal androgen and elevated progesterone levels in women treated for congenital virilizing 21-hydroxylase deficiency. J Clin Endocrinol Metab. (1993) 76:933–6. 8473408 10.1210/jcem.76.4.8473408

[18] Paganini C Radetti G Livieri C Braga V Migliavacca D Adami S. Height, bone mineral density and bone markers in congenital adrenal hyperplasia. Horm Res. (2000) 54:164–8. 10.1159/000053253 11416232

[19] Völkl TMK Simm D Körner A Rascher W Kiess W Kratzsch J. Does an altered leptin axis play a role in obesity among children and adolescents with classical congenital adrenal hyperplasia due to 21-hydroxylase deficiency? Eur J Endocrinol. (2009) 160:239–47. 10.1530/EJE-08-0770 19004982

[20] King JA Wisniewski AB Bankowski BJ Carson KA Zacur HA Migeon CJ. Long-term corticosteroid replacement and bone mineral density in adult women with classical congenital adrenal hyperplasia. J Clin Endocrinol Metab. (2006) 91:865–9. 10.1210/jc.2005-0745 16278269

[21] Roche EF Charmandari E Dattani MT Hindmarsh PC. Blood pressure in children and adolescents with congenital adrenal hyperplasia (21-hydroxylase deficiency): a preliminary report. Clin Endocrinol. (2003) 58:589–96. 10.1046/j.1365-2265.2003.01757.x 12699440

[22] Bonfig W Roehl FW Riedl S Dörr HG Bettendorf M Brämswig J. Blood pressure in a large cohort of children and adolescents with classic adrenal hyperplasia (CAH) due to 21-hydroxylase deficiency. Am J Hypertens. (2016) 29:266–72. 10.1093/ajh/hpv087 26071487

[23] Mooij CF van Herwaarden AE Sweep FCGJ Roeleveld N de Korte CL Kapusta L. Cardiovascular and metabolic risk in pediatric patients with congenital adrenal hyperplasia due to 21 hydroxylase deficiency. J Pediatr Endocrinol Metab. (2017) 30:957–66. 10.1515/jpem-2017-0068 28787274

[24] Amr NH Ahmed AY Ibrahim YA. Carotid intima media thickness and other cardiovascular risk factors in children with congenital adrenal hyperplasia. J Endocrinol Invest. (2014) 37:1001–8. 10.1007/s40618-014-0148-8 25112902

[25] Subbarayan A Dattani MT Peters CJ Hindmarsh PC. Cardiovascular risk factors in children and adolescents with congenital adrenal hyperplasia due to 21-hydroxylase deficiency. Clin Endocrinol. (2014) 80:471–7. 10.1111/cen.12265 23751160 PMC4204515

[26] Isguven P Arslanoglu I Mesutoglu N Yildiz M Erguven M. Bioelectrical impedance analysis of body fatness in childhood congenital adrenal hyperplasia and its metabolic correlates. Eur J Pediatr. (2008) 167:1263–8. 10.1007/s00431-007-0665-y 18204937

[27] Janus D Wójcik M Tyrawa K Janeczko M Bik-Multanowski M Fijorek K. Circadian blood pressure profiles and ambulatory arterial stiffness index in children and adolescents with congenital adrenal hyperplasia due to 21-hydroxylase deficiency in relation to their genotypes. Neuroendocrinol Lett. (2017) 38:509–18. 29369603

[28] Williams RM Deeb A Ong KK Bich W Murgatroyd PR Hughes IA. Insulin sensitivity and body composition in children with classical and nonclassical congenital adrenal hyperplasia. Clin Endocrinol. (2010) 72:155–60. 10.1111/j.1365-2265.2009.03587.x 19508608

[29] Abd El Dayem SM Anwar GM Salama H Kamel AF Emara N Bone mineral density, bone turnover markers, lean mass, and fat mass in Egyptian children with congenital adrenal hyperplasia. Arch Med Sci. (2010) 6:104–10. 10.5114/aoms.2010.13516 22371729 PMC3278952

[30] Marra AM Improda N Capalbo D Salzano A Arcopinto M De Paulis A. Cardiovascular abnormalities and impaired exercise performance in adolescents with congenital adrenal hyperplasia. J Clin Endocrinol Metab. (2015) 100:644–52. 10.1210/jc.2014-1805 25405496

[31] Ariyawatkul K Tepmongkol S Aroonparkmongkol S Sahakitrungruang T. Cardio-metabolic risk factors in youth with classical 21-hydroxylase deficiency. Eur J Pediatr. (2017) 176:537–45. 10.1007/s00431-017-2875-2 28224294

[32] Halper A Sanchez B Hodges JS Kelly AS Dengel D Nathan BM. Bone mineral density and body composition in children with congenital adrenal hyperplasia. Clin Endocrinol. (2018) 88:813–19. 10.1111/cen.13580 29460378 PMC5980722

[33] Kim MS Ryabets-Lienhard A Dao-Tran A Mittelman SD Gilsanz V Schrager SM. Increased abdominal adiposity in adolescents and young adults with classical congenital adrenal hyperplasia due to 21-hydroxylase deficiency. J Clin Endocrinol Metab. (2015) 100:E1153–9. 10.1210/jc.2014-4033 26062016 PMC4524992

[34] Matsubara Y Ono M Miyai K Takizawa F Takasawa K Onishi T. Longitudinal analysis of growth and body composition of Japanese 21-OHD patients in childhood. Endocr J. (2013) 60:149–54. 10.1507/endocrj.EJ12-0123 23018978

[35] Takishima S Nakajima K Nomura R Tsuji-Hosokawa A Matsuda N Matsubara Y. Lower body weight and BMI at birth were associated with early adiposity rebound in 21-hydroxylase deficiency patients. Endocr J. (2016) 63:983–90. 10.1507/endocrj.EJ16-0194 27545660

[36] Charmandari E Weise M Bornstein SR Eisenhofer G Keil MF Chrousos GP. Children with classic congenital adrenal hyperplasia have elevated serum leptin concentrations and insulin resistance: potential clinical implications. J Clin Endocrinol Metab. (2002) 87:2114–20. 10.1210/jcem.87.5.8456 11994350

[37] Harrington J Pe-a AS Gent R Hirte C Couper J. Adolescents with congenital adrenal hyperplasia because of 21-hydroxylase deficiency have vascular dysfunction. Clin Endocrinol. (2012) 76:837–42. 10.1111/j.1365-2265.2011.04309.x 22145701

[38] Zimmermann A Grigorescu-Sido P AlKhzouz C Patberg K Bucerzan S Schulze E. Alterations in lipid and carbohydrate metabolism in patients with classic congenital adrenal hyperplasia due to 21-hydroxylase deficiency. Horm Res Paediatr. (2010) 74:41–9. 10.1159/000313368 20395657

[39] Saygili F Oge A Yilmaz C. Hyperinsulinemia and insulin insensitivity In women with nonclassical congenital adrenal hyperplasia due to 21-hydroxylase deficiency: the relationship between serum leptin levels and chronic hyperinsulinemia. Horm Res. (2005) 63:270–4. 10.1159/000086363 15956788

[40] Bayraktar F Dereli D Ozgen AG Yilmaz C. Plasma homocysteine levels in polycystic ovary syndrome and congenital adrenal hyperplasia. Endocr J. (2004) 51:601–8. 10.1507/endocrj.51.601 15644580

[41] Speiser PW Serrat J New MI Gertner JM. Insulin insensitivity in adrenal hyperplasia due to nonclassical steroid 21-hydroxylase deficiency. J Clin Endocrinol Metab. (1992) 75:1421–4. 1464643 10.1210/jcem.75.6.1464643

[42] Wasniewska M Balsamo A Valenzise M Manganaro A Faggioli G Bombaci S Increased large artery intima media thickness in adolescents with either classical or non-classical congenital adrenal hyperplasia. J Endocrinol Invest. (2013) 36:12–5. 10.1007/BF03346751 22189488

[43] Akyürek N Atabek ME Eklioglu BS Alp H. Ambulatory blood pressure and subclinical cardiovascular disease in patients with congenital adrenal hyperplasia: a preliminary report. J Clin Res Pediatr Endocrinol. (2015) 7:13–8. 10.4274/jcrpe.1658 25800471 PMC4439887

[44] Botero D Arango A Danon M Lifshitz F. Lipid profile in congenital adrenal hyperplasia. Metabolism. (2000) 49:790–3. 10.1053/meta.2000.6261 10877208

[45] Nebesio TD Eugster EA. Observation of hypertension in children with 21-hydroxylase deficiency. A preliminary report. Endocrine. (2006) 30:279–82. 10.1007/s12020-006-0005-4 17526939

[46] Völkl TMK Simm D Dötsch J Rascher W Dörr HG. Altered 24-hour blood pressure profiles in children and adolescents with classical congenital adrenal hyperplasia due to 21-hydroxylase deficiency. J Clin Endocrinol Metab. (2006) 91:4888–95. 10.1210/jc.2006-1069 17003094

[47] Hoepffner W Herrmann A Willgerodt H Keller E. Blood pressure in patients with congenital adrenal hyperplasia due to 21-hydroxylase deficiency. J Pediatr Endocrinol Metab. (2006) 19:705–11. 10.1515/JPEM.2006.19.5.705 16789637

[48] Völkl TM Simm D Körner A Kiess W Kratzsch J Dörr HG. Adiponectin levels are high in children with classic congenital adrenal hyperplasia (CAH) due to 21-hydroxylase deficiency. Acta Paediatr. (2009) 98:885–91. 10.1111/j.1651-2227.2009.01231.x 19236311

[49] Metwalley KA Farghaly HS Sherief T Left ventricular dysfunction and subclinical atherosclerosis in children with classic congenital adrenal hyperplasia: a single-center study from upper Egypt. Eur J Pediatr. (2016) 175:405–12. 10.1007/s00431-015-2634-1 26390869

[50] Metwalley KA Farghaly HS Abdelhamid A Homocysteine level in children with classic congenital adrenal hyperplasia: relationship to carotid intimal wall thickness and left ventricular function. Horm Res Paediatr. (2018) 12:1–8. 10.1159/000492900 30317242

[51] Sartorato P Zulian E Benedini S Mariniello B Schiavi F Bilora F. Cardiovascular risk factors and ultrasound evaluation of intima-media thickness at common carotids, carotid bulbs, and femoral and abdominal aorta arteries in patients with classic congenital adrenal hyperplasia due to 21-hydroxylase deficiency. J Clin Endocrinol Metab. (2007) 92:1015–18. 10.1210/jc.2006-1711 17200174

[52] Rodrigues TM Barra CB Santos JL Goulart EM Ferreira AV Silva IN. Cardiovascular risk factors and increased carotid intima-media thickness in young patients with congenital adrenal hyperplasia due to 21-hydroxylase deficiency. Arch Endocrinol Metab. (2015) 59:541–7. 10.1590/2359-3997000000119 26677089

[53] Özdemir R Korkmaz HA Küçük M Karadeniz C Meşe T Özkan B. Assessment of early atherosclerosis and left ventricular dysfunction in children with 21-hydroxylase deficiency. Clin Endocrinol. (2017) 86:473–79. 10.1111/cen.13275 27905124

[54] Tony Nengom J Sap Ngo Um S Chelo D Mbono Betoko R Boombhi J Mouafo Tambo F. Assessment of cardiac function in children with congenital adrenal hyperplasia: a case control study in Cameroon. BMC Pediatr. (2017) 17:109. 10.1186/s12887-017-0862-4 28427378 PMC5399398

[55] Minetto MA Lanfranco F Motta G Allasia S Arvat E D'Antona G. Steroid myopathy: some unresolved issues. J Endocrinol Invest. (2011) 34:370–75. 10.1007/BF03347462 21677507

[56] Ubertini G Bizzarri C Grossi A Gimigliano F Ravà L Fintini D. Blood pressure and left ventricular characteristics in Young Patients with Classical Congenital Adrenal Hyperplasia due to 21-Hydroxylase Deficiency. Int J Pediatr Endocrinol. (2009) 2009:383610. 10.1186/1687-9856-2009-383610 20169124 PMC2821643

[57] Mooij CF Pourier MS Weijers G de Korte CL Fejzic Z Claahsen-van der Grinten HL. Cardiac function in paediatric patients with congenital adrenal hyperplasia due to 21 hydroxylase deficiency. Clin Endocrinol. (2018) 88:364–71. 10.1111/cen.13529 29230843

[58] Weise M1 Mehlinger SL Drinkard B Rawson E Charmandari E Hiroi M. Patients with classic congenital adrenal hyperplasia have decreased epinephrine reserve and defective glucose elevation in response to high-intensity exercise. J Clin Endocrinol Metab. (2004) 89:591–97. 10.1210/jc.2003-030634 14764767

[59] Green-Golan L Yates C Drinkard B VanRyzin C Eisenhofer G Weise M. Patients with classic congenital adrenal hyperplasia have decreased epinephrine reserve and defective glycemic control during prolonged moderate-intensity exercise. J Clin Endocrinol Metab. (2007) 92:3019–24. 10.1210/jc.2007-0493 17535996

[60] Merke DP Chrousos GP Eisenhofer G Weise M Keil MF Rogol AD. Adrenomedullary dysplasia and hypofunction in patients with classic 21-hydroxylase deficiency. N Engl J Med. (2000) 343:1362–8. 10.1056/NEJM200011093431903 11070100

